# Editorial: Type I and Type III Interferon Immune Responses in Asthma

**DOI:** 10.3389/fimmu.2021.826363

**Published:** 2022-01-18

**Authors:** Susetta Finotto, Tuomas Jartti, Sebastian L. Johnston

**Affiliations:** ^1^ Department of Molecular Pneumology, Department of Anaesthesiology, Friedrich-Alexander-Universität Erlangen-Nürnberg, Erlangen, Germany; ^2^ PEDEGO Research Unit, Medical Research Center, University of Oulu, Oulu, Finland; ^3^ Department of Pediatrics and Adolescent Medicine, Oulu University Hospital, Oulu, Finland; ^4^ Department of Pediatrics and Adolescent Medicine, University of Turku and Turku University Hospital, Turku, Finland; ^5^ National Heart and Lung Institute, Imperial College London, London, United Kingdom

**Keywords:** allergic asthma, air pollution, rhinovirus, children, interferons

Asthma is a chronic inflammatory disease of the airways and causes a major global health burden affecting over 260 million people. It is the most common chronic condition in children, which principally manifests as allergic asthma, a pathological immune response to otherwise innocuous agents (1). Childhood asthma and allergy develop from the interaction between the genetic background of the child and early pre- and post-natal environmental exposures which also influence the maturation of the immune system (2, 3). Air pollution is one of the greatest environmental risks to health in general and to respiratory diseases in particular (4). Virus infections, especially rhinovirus (RV) infections, on the other hand, are significant triggers of asthma attacks (5, 6).

Interferons (IFNs) have a major role in mediating early antiviral responses—especially type I (IFN-α/β) and type III (IFN-λs) IFNs. High virus infection rates were detected in acute wheezing episodes and exacerbations of childhood asthma (up to 100% and 90% respectively). In addition, an association between RV-induced early wheezing episodes and subsequent development of school-age asthma has been reported (odds ratio up to 45 in subjects with aeroallergen sensitization). Altogether these findings have led to the hypothesis that impaired IFN responses may actually have an important role in the development of asthma (6–8). In agreement, knockout mouse models have demonstrated the importance of IFN-α/β in controlling virus replication (9), while studies with IFN-λs report that they suppress both virus replication (10) and allergic airway inflammation (11).

In this topic Makrinioti et al. discussed how clinical data are contradictory on the role of IFNs in asthma development after bronchiolitis. The issue may be partly technical and related to the sampling site (upper *vs* lower airway) as well as timing of sampling in relation to the onset of infection.

Current thinking is that people with wheezing/asthma that is not optimally controlled have deficient and delayed early IFN responses upon virus infection, which leads to greater early virus loads, which then drive subsequent exacerbation severity. In addition, this dysregulated early IFN response leading to greater early virus loads therefore results in subsequent greater IFN concentrations when measured later during virus-induced wheezing episodes (12).

The integrity of the epithelial barrier is also of great importance (13). Viruses, antigens and other environmental particles represent a continuous insult for the respiratory epithelium. A healthy respiratory epithelium fends off those triggers by limiting their cellular and peri-cellular entrance. This is achieved by maintaining an integrity at the cell to cell level. In fact, there are ultrastructural components known as tight junctions that contribute to effective cell-cell epithelial connections that can be disrupted by insults such as infections and allergic airway inflammation (14).

These tight junctions are connected to components of the cytoskeleton, which, along with Toll-like Receptor (TLR) sensor mechanisms, contribute to selective epithelial transmigration, thus constituting a tight barrier. In fact, continuous insults of the respiratory epithelium like virus infections result in damage to these inter-epithelial cell tight junctions thus impairing the integrity of the epithelial barrier to external insults. These environmental triggers are also recognized by resident airway mucosal dendritic cells (DCs). Antigen presentation by DCs leads to an initial activation and expansion of antigen-specific T cells. Alveolar macrophages (AMs) contribute to mucosal homeostasis *via* phagocytosis and sequestration of incoming molecular triggers. Both DCs and AMs can produce anti-viral IFNs, as well as the bronchial epithelial cells themselves (1, 10) (**Figure 1**).

**Figure 1:**
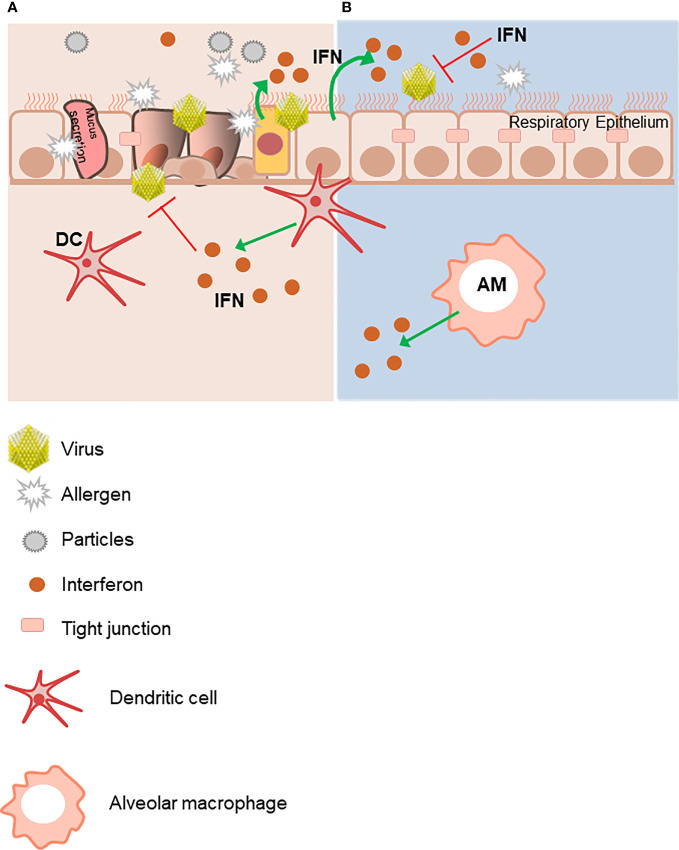
The integrity of the epithelial respiratory barrier is important to protect from environmental triggers. **(A)** Innocuous antigens, environmental particles and viruses damage the integrity of the mucosal epithelium barrier. The respiratory mucosal epithelium barrier must facilitate selective transport as well as restrict free exchange across the para-cellular spaces. These functions are facilitated by the tight junctions (TJs), which are molecular structures between cells allowing closest cell to cell contact. They regulate the permeability of ions, (macro) molecules and cells *via* the paracellular pathway. Virus-infected epithelial cells are an important immediate source of IFNs, as well as later production by dendritic cells (DCs) and alveolar macrophages (AMs). **(B)** In contrast, lack of exogenous triggers maintains an intact respiratory epithelial barrier. This figure was created using Servier Medical Art templates, which are licensed under a Creative Commons Attribution 3.0 Unported License; https://smart.servier.com.

In this topic, we focused on the immune response to RV infection, in the presence or not of air pollution. Here we asked if a polluted microenvironment would promote RV infection and reduce IFN antiviral immune responses in children. The review from Yang et al. summarizes recent literature on the expression of type I IFNs in association with RV infection in asthma. Here the mechanism of RV infection in asthma and the associated IFN response, especially the type I response, were interpreted. The authors pointed out their view on the important functional role of type I IFNs in response to RV infection (15). Additionally, the authors gave readers an overview on the cellular mechanisms in innate/adaptive immunity and cytokine secretion during RV infection based on recently advanced studies. This review opens the avenue to new therapeutic applications in the treatment of asthma and asthma exacerbations associated with RV infection.

Consistent with a key role of type I IFNs in antiviral immune responses, the manuscript from Bonato et al. in this topic addressed experimentally the important issue of the impact of air pollution in the airways of children with asthma. Here, the authors found that acute exposure to PM10 and NO_2_ was negatively associated with RV-A16-induced IFN-β mRNA. No significant associations were detected between IFN-λ mRNA and NO_2_ or PM10. Increasing levels of NO_2_ (but not PM10) were found to be associated with increased RV-A16 replication. This manuscript thus indicates that air pollution could exacerbate childhood asthma due to inhibition of type I IFN anti-viral immune responses in the airway epithelial barrier.

Bronchiolitis is usually defined as a virus-induced inflammation of small bronchioles and their surrounding tissue in infants. The airway inflammation consequently causes airway edema, mucus hyper-production and bronchial smooth muscle contraction, making it more difficult to breathe (5, 6). In this issue, Makrinioti et al. discuss the role of IFNs in driving susceptibility to asthma following bronchiolitis. Innate type I & III IFNs may play an important role in regulating the development of asthma following bronchiolitis in infancy. There are, however, several knowledge gaps, and future research can help address these. The main research gaps include the understanding of IFN kinetics following respiratory viral infection in children of different ages and the use of IFNs as markers of bronchiolitis severity or susceptibility to asthma. Future research can highlight whether IFNs could be used as treatment in severe bronchiolitis with potential disease modifying effect. Connecting methodological development (mathematical modeling) with biological data could further drive problem solving in asthma development following bronchiolitis.

Finally, in the review from Krammer et al., the authors summarize knowledge on the expression, regulation and function of IFN-λs. An important part is also the regulation of interferon-λ-receptor (IFN-λR) by TLR agonists like R848. Krammer et al. discuss the interesting possibility to use TLR-7/8 agonists as modulators of IFN-λR expression, to increase responsiveness to IFN-λs. Furthermore, they show the importance of providing further research on soluble forms of IFN-λR and the role of IFN-λs in new infectious diseases like SARS-CoV-2. Taken together, this review provides a comprehensive view on the role of IFN-λs and their receptor during different stages of asthma disease as well as outside the scope of asthma.

In conclusion, this topic highlights the importance of an intact epithelial barrier releasing type I and type III IFNs at the interface between the environment and the child’s inner tissues. Moreover, the effect of type III IFNs can be modulated by antiviral TLR agonists inducing the IFN-λR in peripheral blood cells. Combined with recent literature, these new fields of investigation highlight the possibility to prevent the development and exacerbation of asthma by activating preventive actions by modulating the environment of the child. In addition, in a therapeutic setting, physiologically upregulating type I and III IFNs and their receptors should provide a more rapid and more potent antiviral immune response to protect children from virus-induced wheezing illness. These findings warrant further studies on factors modulating innate IFN responses among young wheezing children who are prone to develop asthma, and thereby, help to improve primary and secondary prevention strategies for asthma.

## Author Contributions

The authors SF, TJ, and SLJ did the drafting of this work and revised it critically for important intellectual content. All authors contributed to the article and approved the submitted version.

## Funding

This work was supported by a grant, awarded to SF, from the Collaborative Research Centre (CRC) 1181- Project B08N (Erlangen), German Research Foundation (DFG) at the University hospital Erlangen,Germany.

## Conflict of Interest

SLJ is an author on patents on the use of inhaled interferons for the treatment of exacerbations of airway diseases.

The remaining authors declare that the research was conducted in the absence of any commercial or financial relationships that could be construed as a potential conflict of interest.

## Publisher’s Note

All claims expressed in this article are solely those of the authors and do not necessarily represent those of their affiliated organizations, or those of the publisher, the editors and the reviewers. Any product that may be evaluated in this article, or claim that may be made by its manufacturer, is not guaranteed or endorsed by the publisher.

## References

[1] Krug J Kiefer A Koelle J Vuorinen T Xepapadaki P Stanic B. TLR7/8 Regulates Type I and Type III Interferon Signalling in Rhinovirus 1b-Induced Allergic Asthma. Eur Respir J (2021) 57. doi: 10.1183/13993003.01562-2020 33303556

[2] Melen E Standl M Gehring U Altug H Anto JM Berdel D Bergstrom A. Air Pollution and IgE Sensitization in 4 European Birth Cohorts-the MeDALL Project. J Allergy Clin Immunol (2021) 147:713–22. doi: 10.1016/j.jaci.2020.08.030 32926877

[3] Le Souef PN. Gene-Environmental Interaction in the Development of Atopic Asthma: New Developments. Curr Opin Allergy Clin Immunol (2009) 9:123–7. doi: 10.1097/ACI.0b013e3283292283 19295429

[4] Velasco RP Appoh E. WHO Global Air Quality Guidelines 2021 Setting Ambitious Goals for Air Quality to Protect Public Health WHO Global Air Quality Guidelines 2021 (2021). Available at: https://cdn.who.int/media/docs/default-source/air-quality-and-health/who-global-aqgs.-afro-presentation-2-nov-2021_final.pdf?sfvrsn=7d2f3da7_5.

[5] Jartti T Bonnelykke K Elenius V Feleszko W. Role of Viruses in Asthma. Semin Immunopathol (2020) 42:61–74. doi: 10.1007/s00281-020-00781-5 31989228 PMC7066101

[6] Read JF Bosco A. Decoding Susceptibility to Respiratory Viral Infections and Asthma Inception in Children. Int J Mol Sci (2020) 21. doi: 10.3390/ijms21176372 PMC7503410 32887352

[7] Turunen R Koistinen A Vuorinen T Arku B Söderlund-Venermo M Ruuskanen O Jartti T. The First Wheezing Episode: Respiratory Virus Etiology, Atopic Characteristics, and Illness Severity. Pediatr Allergy Immunol (2014) 25:796–803. doi: 10.1111/pai.12318 25444257 PMC7167827

[8] Rubner FJ Jackson DJ Evans MD Gangnon RE Tisler CJ Pappas TE. Early Life Rhinovirus Wheezing, Allergic Sensitization, and Asthma Risk at Adolescence. J Allergy Clin Immunol (2017) 139:501–7. doi: 10.1016/j.jaci.2016.03.049 PMC5104680 27312820

[9] Demoor T Petersen BC Morris S Mukherjee S Ptaschinski C De Almeida Nagata DE. IPS-1 Signaling has a Nonredundant Role in Mediating Antiviral Responses and the Clearance of Respiratory Syncytial Virus. J Immunol (2012) 189:5942–53. doi: 10.4049/jimmunol.1201763 PMC3888965 23136205

[10] Contoli M Message SD Laza-Stanca V Edwards MR Wark PAB Bartlett NW. Role of Deficient Type III Interferon-Lambda Production in Asthma Exacerbations. Nat Med (2006) 12:1023–6. doi: 10.1038/nm1462 16906156

[11] Koltsida O Hausding M Stavropoulos A Koch S Tzelepis G Ubel C. IL-28a (IFN-Lambda2) Modulates Lung DC Function to Promote Th1 Immune Skewing and Suppress Allergic Airway Disease. EMBO Mol Med (2011) 3:348–61. doi: 10.1002/emmm.201100142 PMC3377081 21538995

[12] Altman MC Gill MA Whalen E Babineau DC Shao B Liu AH. Transcriptome Networks Identify Mechanisms of Viral and Nonviral Asthma Exacerbations in Children. Nat Immunol (2019) 20:637–51. doi: 10.1038/s41590-019-0347-8 PMC6472965 30962590

[13] Akdis CA. Does the Epithelial Barrier Hypothesis Explain the Increase in Allergy, Autoimmunity and Other Chronic Conditions? Nat Rev Immunol (2021) 21:739–51. doi: 10.1038/s41577-021-00538-7 33846604

[14] Linfield DT Raduka A Aghapour M Rezaee F. Airway Tight Junctions as Targets of Viral Infections. Tissue Barriers (2021) 9:1883965. doi: 10.1080/21688370.2021.1883965 33632074 PMC8078511

[15] Bergauer A Sopel N Kroß B Vuorinen T Xepapadaki P Weiss ST. IFN-Alpha/IFN-Lambda Responses to Respiratory Viruses in Paediatric Asthma. Eur Respir J (2017) 49. doi: 10.1183/13993003.00969-2016

